# Risk and protective factors for interpersonal revictimization in people with post-traumatic stress symptoms: a systematic review

**DOI:** 10.3389/fpsyg.2025.1610030

**Published:** 2025-12-10

**Authors:** Christin Kühner, Inez Verdaasdonk, Carolien Christ, Anneke E. Goudriaan, Kathleen Thomaes, Marleen de Waal

**Affiliations:** 1 Department of Psychiatry, Amsterdam University Medical Centers, Location University of Amsterdam, Amsterdam, Netherlands; 2 Arkin Mental Health Care, Department of Research, Amsterdam, Netherlands; 3 Sinai Centrum, Arkin Mental Health Care, Amstelveen, Netherlands; 4 Amsterdam Institute of Addiction Research (AIAR), Jellinek, Arkin Mental Health Care, Amsterdam, Netherlands; 5 Department of Clinical Psychology, VU University and Amsterdam Public Health Research Institute, Amsterdam, Netherlands; 6 MRC Research Centre, Parnassia Groep, The Hague, Netherlands

**Keywords:** PTSD, revictimization, interpersonal violence, social support, substance use, childhood maltreatment, intimate partner violence

## Abstract

**Introduction:**

People suffering from a psychiatric disorder are at increased risk of being the victim of repeated interpersonal victimization (i.e., revictimization). A psychiatric disorder that has been investigated as both a risk factor and a consequence of interpersonal revictimization is post-traumatic stress disorder (PTSD). However, to date no systematic review has provided an exclusive overview of longitudinally assessed risk or protective factors for interpersonal revictimization in the context of PTSD. With this systematic review, we aim to provide an overview of (a) which facets of PTSD are risk factors for interpersonal revictimization, and (b) which non-PTSD factors are risk or protective factors for interpersonal revictimization?

**Methods:**

Following our pre-registered systematic search of PubMed, APA PsycInfo, PTSDpubs, Web of Science, and Scopus, we screened *N* = 1,286 and included *N* = 16 longitudinal studies.

**Results:**

In the majority of the studies, the overall severity of PTSD symptoms emerged as a risk factor, while the evidence remained mixed for the severity of hyperarousal and negative changes in mood and cognition. We found no evidence that the severity of intrusion, avoidance, or dissociation are risk factors for interpersonal revictimization. For the non-PTSD risk factors, a majority of studies indicated that the severity of prior victimization and drug use were associated with revictimization. The results remained mixed for problematic alcohol use, childhood maltreatment, depression and maladaptive coping. The protective factors, social support and adaptive coping, did not consistently reduce revictimization.

**Discussion:**

PTSD symptom severity was a consistent predictor of revictimization; consequently, reducing PTSD symptom severity as quickly as possible may decrease risk for revictimization. Additional factors to address during intervention are discussed. The study has several limitations, such as the overrepresentation of female participants and the reliance on convenience samples, particularly those involving women residing in shelters. Additionally, there was a disproportionate focus on intimate partner violence (IPV) and sexual abuse as forms of revictimization. Furthermore, we observed considerable heterogeneity in the operationalization and measurement of PTSD (symptoms) and revictimization. Implications of this study are that reducing PTSD symptoms and drug use may decrease risk for future revictimization.

**Systematic review registration:**

https://www.crd.york.ac.uk/prospero/, identifier CRD42023446788.

## 1 Introduction

People with psychiatric disorders are at increased risk of being the victim of interpersonal violence, defined as physical assault, sexual assault, or threats of assault (Maniglio, 2009; De Waal et al., 2017). Whereas the incidence of physical assault, sexual assault or threat of assault in the general population is 23%, this number increases more than two-fold to 58% in psychiatric patients (De Waal et al., 2017). Specifically, psychiatric patients had 14.8 times higher chances of experiencing physical assault in the past year, when compared to the general population (De Waal et al., 2017). Multiple psychiatric disorders, such as substance use disorder, schizophrenia, non-schizophrenia psychoses, bipolar disorder, and personality disorders, were associated with interpersonal victimization (Fazel et al., 2018). Importantly, of those psychiatric patients that are victims of interpersonal violence, the majority (52%) experience multiple types of violence over time, a phenomenon called *revictimization* (Christ et al., 2022).

A specific psychiatric disorder that has been investigated as both a risk factor and a consequence of interpersonal revictimization, is post-traumatic stress disorder (PTSD). PTSD is characterized by symptoms of intrusion, avoidance, negative changes in mood and cognition, and hyperarousal (American Psychiatric Association, 2013). In systematic reviews on the relationship between childhood maltreatment and revictimization in adulthood, PTSD symptoms were identified as both a potential consequence and predictor of (sexual) revictimization, depending on the study under examination (Fereidooni et al., 2023b; Scoglio et al., 2022; Walker and Wamser-Nanney, 2023). Another line of inquiry is whether the experience of revictimization increases the risk for the development of PTSD symptoms. Cividanes et al. (2018) conducted a systematic review on the development of PTSD symptoms in people who experienced only childhood sexual abuse (CSA), only adulthood sexual abuse (ASA), or both. They demonstrated that those who were revictimized (both CSA and ASA) had the highest odds of developing PTSD (Cividanes et al., 2018). Notably, they included mostly cross-sectional studies that retrospectively assessed CSA, meaning that no causal inferences can be drawn whether exposure to revictimization caused PTSD. Given the multitude of studies that investigate PTSD symptoms and revictimization, an examination of the theoretical frameworks linking PTSD symptoms and revictimization may advance our understanding of their interrelationships.

Multiple theoretical frameworks have been proposed to explain the link between PTSD symptoms and revictimization. First, the ‘post-traumatic stress symptom model’ of revictimization was put forth, according to which post-traumatic stress symptoms increase the risk of revictimization after childhood maltreatment (Walker and Wamser-Nanney, 2023). In this model the authors postulate that post-traumatic stress symptoms can manifest as difficulties in emotion regulation, proneness to anger, maladaptive coping, and dissociation, which are in turn related to revictimization. This model is based on a review of the literature and a synthesis of the empirical findings thus far (Walker and Wamser-Nanney, 2023). Second, the ‘evidence-based model’ for revictimization after childhood maltreatment was formulated (Fereidooni et al., 2023a). The model was based on the results of structural equation modelling on a large dataset in female college students. The authors examined which factors were higher order mediators for the relationship between exposure to childhood maltreatment and revictimization in adulthood, and identified PTSD symptoms, trauma load, peri-traumatic dissociation, drug use, and loneliness as higher order mediators (Fereidooni et al., 2023a). Third, the ‘betrayal trauma theory’ (BTT) posits that exposure to childhood maltreatment is associated with a diminished ability to detect danger cues in relationships, which can put individuals at increased risk of revictimization (Freyd, 1994; Deprince, 2005). Studies have provided evidence that people exposed to high levels of betrayal trauma during childhood, i.e., trauma perpetrated by a trusted person/caregiver, reported more betrayal trauma in adulthood, a form of revictimization (Mackelprang et al., 2014). In the same study, a higher cumulative exposure to interpersonal and non-interpersonal trauma levels in adulthood was associated with negative mental health outcomes, such as symptoms of depression and PTSD. In sum, all three models state PTSD symptoms as a mediator for revictimization.

Despite multiple theoretical frameworks and growing evidence of associations between PTSD and revictimization, existing reviews remain limited in generalizability due to their emphasis on specific victimization types and developmental stages, and the lack of longitudinal research addressing temporal dynamics in these associations. To date, no pre-registered systematic review has provided a broad overview of factors associated longitudinally with revictimization in the context of PTSD (symptoms).

We aim to overcome the limitations of earlier reviews by conducting a systematic review focused exclusively on longitudinal studies and risk and protective factors. This approach allows us, in contrast to earlier reviews, to draw conclusions about the role of PTSD symptoms as a potential risk factor for revictimization. Our first aim was to clarify the role of PTSD symptoms as risk factors for revictimization, by providing an overview of the findings of all longitudinal studies that have examined this relationship. The second aim was to provide an overview of which non-PTSD risk or protective factors for revictimization were investigated in the context of PTSD symptoms. The resulting knowledge is of clinical relevance, given the vulnerability of psychiatric populations for revictimization. Further, the results may be used to identify targets for the prevention of interpersonal revictimization by providing a theoretical overview of risk and protective factors.

## 2 Methods

We conducted a pre-registered systematic search of the literature in accordance to the guidelines described in the PRISMA protocol (Page et al., 2021). The pre-registration was posted to the online platform PROSPERO, which is specifically set-up for systematic reviews (PROSPERO). We used the following MeSH-terms “Stress Disorders, Traumatic” and “Adult Survivors of Child Abuse” in combination with a range of title, abstract, and author supplied keywords for “child abuse,” “revictimization,” and “victimization.” For a full overview of the search terms per database, see Supplementary material. We searched for the combination of these terms in PubMed, APA PsycInfo, PTSDpubs, Web of Science, and Scopus in September 2024.

### 2.1 Selection of the literature

**Figure 1:**
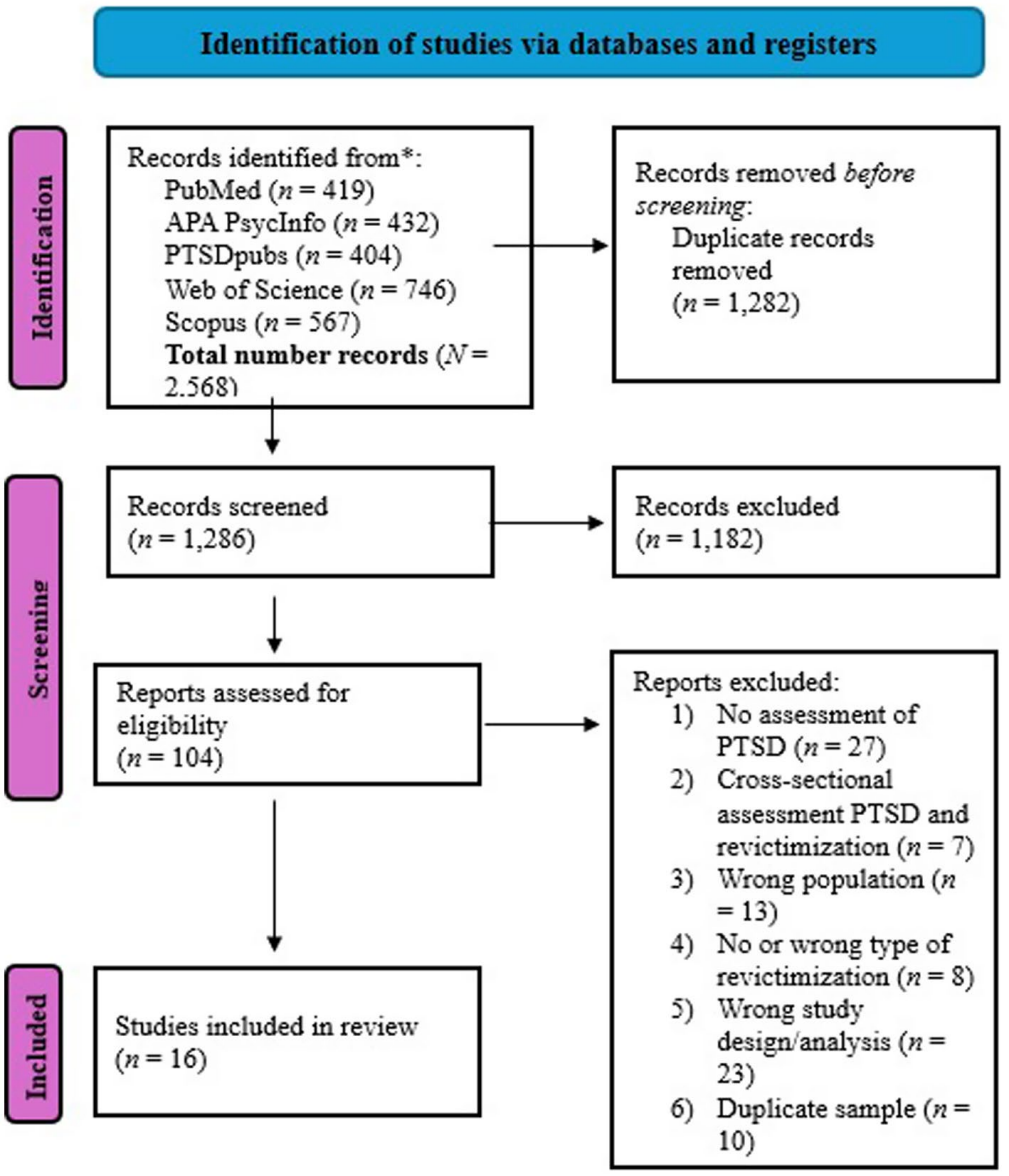
PRISMA flowchart of the systematic literature search.

We applied the following inclusion criteria for the publications that resulted from our search: (1) adult human sample with a mean age of 18 years or older, (2) assessment of PTSD symptoms (either cluster-scores or total score) according to DSM-criteria, (3) assessment of interpersonal revictimization, i.e., physical assault, and/or sexual assault, and/or threat of assault, and/or emotional violence, (4) published between 1980 and 2023 since PTSD was introduced as a diagnosis in 1980, (5) published in a peer-reviewed journal, (6) longitudinal study design, (7) assessment of PTSD symptoms preceded assessment of interpersonal revictimization, and (8) the entire sample was victimized at baseline OR the authors included subgroup analyses for those who were victimized at baseline. Exclusion criteria were: (1) single case studies, (2) studies with fewer than *N* = 20 participants (Simmons et al., 2011), (3) meta-analyses or systematic reviews, (4) intervention studies, and (5) published in a language other than English. The first author screened all articles and KT (97% interrater agreement), MdW (98% interrater agreement), and AG (98% interrater agreement) each screened a third of all articles. If there was a disagreement in the inclusion decision that could not be resolved, one of the other screeners was consulted for a discussion (*n* = 1). All interrater agreements refer to percentages of agreement. The first author conducted the full-text screening and data extraction with IV (90% interrater agreement) (see Figure 1).

### 2.2 Data extraction

We extracted the following study data: sample size, year of publication, type of sample, type of revictimization incident, identified risk and protective factors, and non-significant predictors. Reporting non-significant factors is valuable given the inflation of effect estimates when the literature reports only on those studies that found a significant effect for a specific risk/protective factor (Franco et al., 2014). If multiple papers were based on the same sample, we chose the paper that provided the most direct answer to our research question (i.e., Which facets of PTSD are associated with revictimization? Which non-PTSD factors are associated with revictimization?).

### 2.3 Inclusion of risk and protective factors

We were most interested in the role of PTSD symptoms as risk factors for interpersonal revictimization. We investigated both the overall symptom severity, and where applicable, the role of symptom clusters. Given the changes in PTSD symptom clusters from DSM-IV to DSM-5, we decided to group the symptom cluster “numbing” into “negative changes in mood and cognition” and to rename “re-experiencing” into “intrusions,” wherever applicable. This is in line with official guidelines on the transition from DSM-IV to DSM-5 (American Psychiatric Association, 2013). In our text/figures we therefore use the DSM-5 jargon; however, in the overview of all included studies (Table 1), we kept the original wording of the included articles for transparency. See Table 2 for an overview of the definitions of all concepts included in the systematic review.

**Table 1:** Overview of studies and main findings included in the systematic review.

Author (year)	Sample size (% female)	Type of sample	Type of revictimization	Significant risk factors of revictimization	Significant protective factors of revictimization	Non-significant factors of revictimization
Aakvaag et al. (2019)	505 (60.0%)	Convenience (young adults with childhood violence history)	Physical assault IPV Sexual assault	Trauma-related shame Problematic alcohol use		Trauma-related guilt Social support Severity of post-traumatic stress symptoms Any number of negative family background factors
Amstadter et al. (2011)	1,753 (49.8%)	General population	Physical assault	African American ethnic status (compared to Caucasian) Male gender History of CPA Witnessing violence Problematic drug use		Age Family income History of CSA PTSD severity Problematic alcohol use Family alcohol problem Family drug problem
Babcock and Deprince (2013)	192 (100%)	Battered women	IPV	Childhood betrayal trauma Depression PTSD severity Unemployment		Dissociation No. of children < age 13 IPV incident severity
Cascardi and Avery-Leaf (2019)	53 (100%)	Convenience (college students)	IPV	PTSD hyperarousal symptoms More frequent emotional abuse Threatening behavior		Prior victimizations PTSD re-experiencing symptoms PTSD avoidance symptoms PTSD numbing symptoms Same partner at baseline and follow-up
Cole et al. (2008)	632 (100%)	Convenience (women with DVO)	IPV	Cumulative lifetime victimization index No. of months involved with new partner (since T0^a^) Problematic drug use (in year before T0)		Age Area (sociodemographic factor) Highest level of education Annual income Social support Social obstruction Daily hassles Depression PTSD (meeting study criteria for the diagnosis) Problematic alcohol use (in year before T0)
Dardis et al. (2018)	101 (100%)	Veteran	IPV	Psychological IPV: PTSD severity	Physical and/or sexual IPV: Empowerment	Psychological IPV: Empowerment T0 IPV Physical and/or sexual IPV: PTSD severity T0 IPV
Iverson et al. (2013)	69 (100%)	Women seeking help for IPV	IPV (physical)	T0 physical IPV Disengagement coping	Engagement coping	PTSD hyperarousal symptoms Dissociation
Iverson et al. (2022)	1,839 (100%)	Veteran	IPV	T1^a^ revictimization: T0 IPV T0 PTSD severity T2^a^ revictimization T1 IPV T1 revictimization		Age Combat experience MST
Jaffe et al. (2019)	413 (100%)	Convenience (college students)	Physical assault Emotional/psychological abuse IPV Sexual assault	T0 interpersonal trauma PTSD severity (mediator T0 interpersonal trauma—revictimization) Threat of harm (mediator T0 interpersonal trauma—revictimization)		Self-worth & judgement Reliability & trustworthiness of others
Krause et al. (2006)	329 (100%)	Women seeking help for IPV	IPV	T0 IPV severity PTSD numbing symptoms	Years involved with abusing partner	Child abuse severity (physical & sexual) PTSD re-experiencing symptoms PTSD avoidance symptoms PTSD hyperarousal symptoms
Kuijpers et al. (2012)	156 (100%)	Women seeking help for IPV	IPV	Physical revictimization: Any recent physical IPV victimization Any recent psychological IPV perpetration by victim Psychological revictimization: Any recent psychological IPV victimization		Physical revictimization: Any recent physical IPV perpetration by victim Any recent psychological IPV victimization PTSD re-experiencing symptoms Psychological revictimization: Any recent psychological IPV perpetration by victim Any recent physical IPV victimization PTSD re-experiencing symptoms
Perez et al. (2012)	103 (100%)	Women in battered women shelter	IPV	PTSD severity	Length of shelter stay	
Perez and Johnson (2008)	320 (100%)	Battered women	IPV	PTSD severity		T0 violence severity Help-seeking Social support
Scoglio et al. (2022)	673 (45.7%)	Veteran	Physical assault Sexual assault	Female gender Post-traumatic stress symptom severity Military branch (navy)	Social support during deployment Study visit	Combat exposure Social support post-deployment Problematic alcohol use
Stockdale et al. (2014)	445 (100%)	Convenience (women with DVO)	Sexual assault	PTSD severity (mediator CSA—SH and CNSA—SH)		PTSD severity (mediator IPV-DVO—SH)
Ullman and Najdowski (2011)	555 (100%)	Convenience (women with history of SA)	Sexual assault	PTSD severity	Positive reactions disclosure victimization incident	Negative reactions Characterological self-blame Adaptive coping Maladaptive coping

a Baseline assessments are indicated with T0, and first and second follow-up assessments (with varying time points) are indicated with T1 and T2.

IPV, intimate partner violence; CPA, childhood physical abuse; CSA, childhood sexual abuse; SA, sexual assault PTSD, post-traumatic stress disorder; MST, military sexual trauma; TBI, traumatic brain injury; DVO, domestic violence protective order; SH, sexual harassment; CNSA, childhood non-sexual abuse; IPV-DVO, intimate partner violence by the partner with a domestic violence order against him.

**Table 2:** Definitions of concepts examined.

Concept	Definition
Interpersonal revictimization	Any recurrence of physical assault, sexual assault, threats of assault, emotional violence, or intimate partner violence after a previous incident of interpersonal violence. The revictimization incident can be the same or a different type of violence. The (re-)victimization incidents can also take place within or across different developmental periods (i.e., childhood and/or adulthood).
PTSD symptoms	PTSD is a psychiatric disorder that according to the DSM-5 is characterized by four symptom clusters: intrusion, avoidance, negative changes in mood and cognition, hyperarousal (American Psychiatric Association, 2013). The diagnosis can be specified as “with/without dissociative symptoms.”
Intrusion	One of the symptom clusters of PTSD that describes unwanted memories/flashbacks of the traumatic event. In the DSM-IV this symptom cluster was called “re-experiencing.”
Avoidance	One of the symptom clusters of PTSD that describes avoidance of external or internal stimuli that are associated with the traumatic event.
Negative changes in mood and cognition	One of the symptom clusters of PTSD that refers to negative convictions about the self, world, and others, feelings of guilt, the experience of negative emotions and/or the absence of positive emotions. In the DSM-IV some of these symptoms were grouped under “numbing.”
Hyperarousal	One of the symptom clusters of PTSD that describes hypervigilance, difficulties with concentration and/or sleep.
Dissociation	A symptom that can occur in the context of PTSD but is not necessary for the diagnosis of PTSD. Dissociation refers to a broad spectrum of experiences, from daydreaming to derealization and/or depersonalization.
Childhood maltreatment	Any form of physical, sexual or emotional abuse, and physical or emotional neglect that occurred before age 18.
Victimization severity	The severity of previous violence that was usually assessed at baseline.
Other psychiatric disorders	The only other psychiatric disorder in the included studies, besides symptoms of PTSD, was depression.
Problematic alcohol use	Alcohol use that was associated with either (a) problems with moderation of consumption/stopping or (b) problems with daily functioning.
Problematic drug use	Drug use that was associated with either (a) problems with moderation of consumption/stopping or (b) problems with daily functioning.
Maladaptive coping^1^	Stress reduction through denial or withdrawal.
Adaptive coping^1^	Stress reduction through problem solving and/or approach behavior.
Social support^1^	Either (a) having a person in one’s social network that can help with daily hassles, provide emotional support or (b) positive reactions to disclosure of sexual assault.

1 The definitions of maladaptive coping, adaptive coping and social support were based on the articles that included these factors (Cole et al., 2008; Aakvaag et al., 2019; Ullman and Najdowski, 2011).

### 2.4 Risk of bias

We employed the risk of bias assessment tool for the prevalence of mental health disorders (RoB-PrevMH) to quantify risk of bias in the included studies (Tonia et al., 2023). The RoB-PrevMH tool consists of three subscales: sampling frame bias, responder bias, and information bias. Sampling frame bias refers to whether the study sample is an accurate representation of the target population. Responder bias refers to which proportion of all people who were approached participated in the study. Information bias refers to whether a validated instrument was used to assess the outcome of interest (i.e., revictimization). The RoB-PrevMH has been developed specifically for observational studies in the field of mental health. We independently assessed the risk of bias with two assessors in the research team (CK and CC). After the individual assessments, scores were compared and discussed whenever they did not match (interrater agreement = 0.98).

## 3 Results

### 3.1 Overview of studies

Table 1 provides an overview of the studies included in this systematic review. All *N* = 16 studies that were included employed a longitudinal design, whereby PTSD symptoms were measured before interpersonal revictimization. The included sample sizes range from *n* = 53 to *n* = 1839, resulting in an overall sample size of *N* = 8,138 for the current study. The proportion of females included in the sample range from 45.7–100%, with *n* = 13 studies including only females.

The types of interpersonal revictimization most commonly studied were (not mutually exclusive): intimate partner violence (IPV; *n* = 11), sexual assault (*n* = 5), physical assault (*n* = 4), and emotional violence (*n* = 1). Notably, measures of IPV commonly consist of subscales for sexual assault, physical assault, and emotional violence. Only six studies studied one of the protective factors defined above (Aakvaag et al., 2019; Cole et al., 2008; Iverson et al., 2013; Perez and Johnson, 2008; Scoglio et al., 2022; Ullman and Najdowski, 2011).

### 3.2 PTSD symptoms as risk factors for interpersonal revictimization

**Figure 2:**
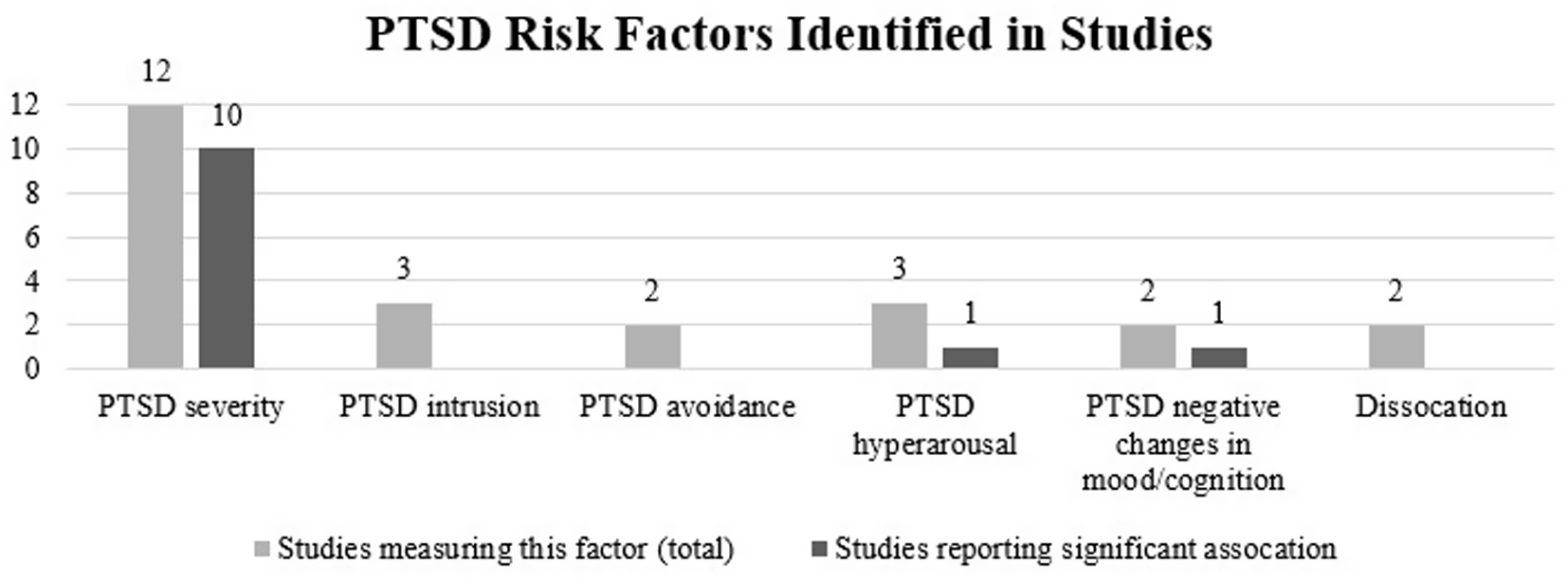
PTSD risk factors for interpersonal revictimization in the included studies (*N* = 16). PTSD severity refers to the overall severity of PTSD symptoms. The other PTSD risk factors refer to the severity of the symptom cluster scores (i.e., intrusion, avoidance, hyperarousal, negative changes in mood/cognition) and dissociation.

Of the included sixteen studies, twelve measured the overall severity of PTSD, three studies assessed intrusion, two studies assessed avoidance, three studies assessed hyperarousal, two studies assessed negative changes in mood and cognition, and two assessed dissociation. Ten out of twelve studies found that the overall severity of PTSD predicted some form of interpersonal revictimization (Aakvaag et al., 2019; Amstadter et al., 2011; Babcock and Deprince, 2013; Cole et al., 2008; Dardis et al., 2018; Iverson et al., 2022; Jaffe et al., 2019; Perez et al., 2012; Perez and Johnson, 2008; Scoglio et al., 2022; Stockdale et al., 2014; Ullman and Najdowski, 2011). Notably, the study by Dardis et al. (2018) found that PTSD symptom severity significantly predicted psychological IPV revictimization but not physical or sexual IPV revictimization. Similarly, Stockdale et al. (2014) demonstrated that PTSD symptom severity significantly predicted sexual harassment after childhood sexual assault, but not sexual harassment after IPV. Only the studies by Amstadter et al. (2011) and Cole et al. (2008) reported no association between the severity of PTSD and revictimization. In terms of symptom clusters, none of three studies found that symptoms of intrusion predicted interpersonal revictimization (Cascardi and Avery-Leaf, 2019; Krause et al., 2006; Kuijpers et al., 2012). None of the two studies that examined the relationship between avoidance symptoms and interpersonal revictimization found a significant association (Cascardi and Avery-Leaf, 2019; Krause et al., 2006). One of three studies demonstrated that hyperarousal symptoms predicted interpersonal revictimization (Cascardi and Avery-Leaf, 2019). One of two studies found that negative changes in mood and cognition significantly predicted interpersonal revictimization (Krause et al., 2006). None of the two studies that examined the relationship between dissociation and interpersonal revictimization reported a significant association (Babcock and Deprince, 2013; Iverson et al., 2013). See Figure 2 for an overview of the PTSD risk factors that were examined.

### 3.3 Non-PTSD risk factors for interpersonal revictimization

**Figure 3:**
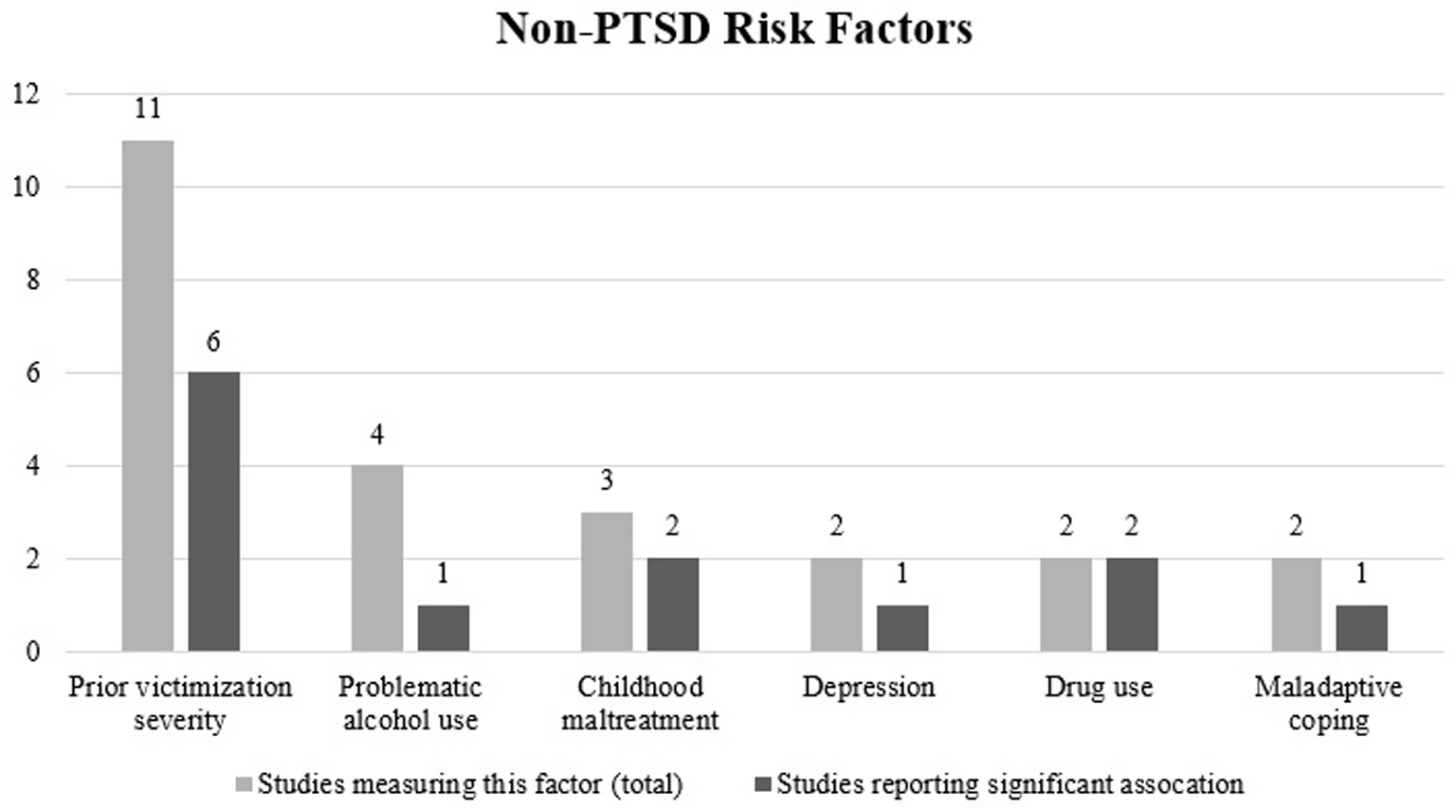
Non-PTSD risk factors for interpersonal revictimization in the included studies (*N* = 16).

The most commonly studied (non-PTSD) risk factor for interpersonal revictimization was victimization severity at baseline, which significantly predicted interpersonal revictimization in six out of eleven studies (Amstadter et al., 2011; Cole et al., 2008; Iverson et al., 2013; Iverson et al., 2022; Jaffe et al., 2019; Krause et al., 2006). One of four studies found that problematic alcohol use significantly predicted interpersonal revictimization (Aakvaag et al., 2019). Two of three studies reported that childhood maltreatment was a significant predictor for interpersonal revictimization (Amstadter et al., 2011; Babcock and Deprince, 2013). Notably, the study by Amstadter et al. (2011) demonstrated that a history of childhood *physical* abuse predicted interpersonal revictimization, while a history of childhood *sexual* abuse did not. One of two studies demonstrated that depression predicted interpersonal revictimization (Babcock and Deprince, 2013). Both studies that examined the relationship between drug use (other than alcohol) and interpersonal revictimization demonstrated that drug use predicted interpersonal revictimization (Amstadter et al., 2011; Cole et al., 2008). One of two studies reported that maladaptive coping predicted interpersonal revictimization (Iverson et al., 2013). See Figure 3 for an overview of the risk factors.

### 3.4 Protective factors against interpersonal revictimization

**Figure 4:**
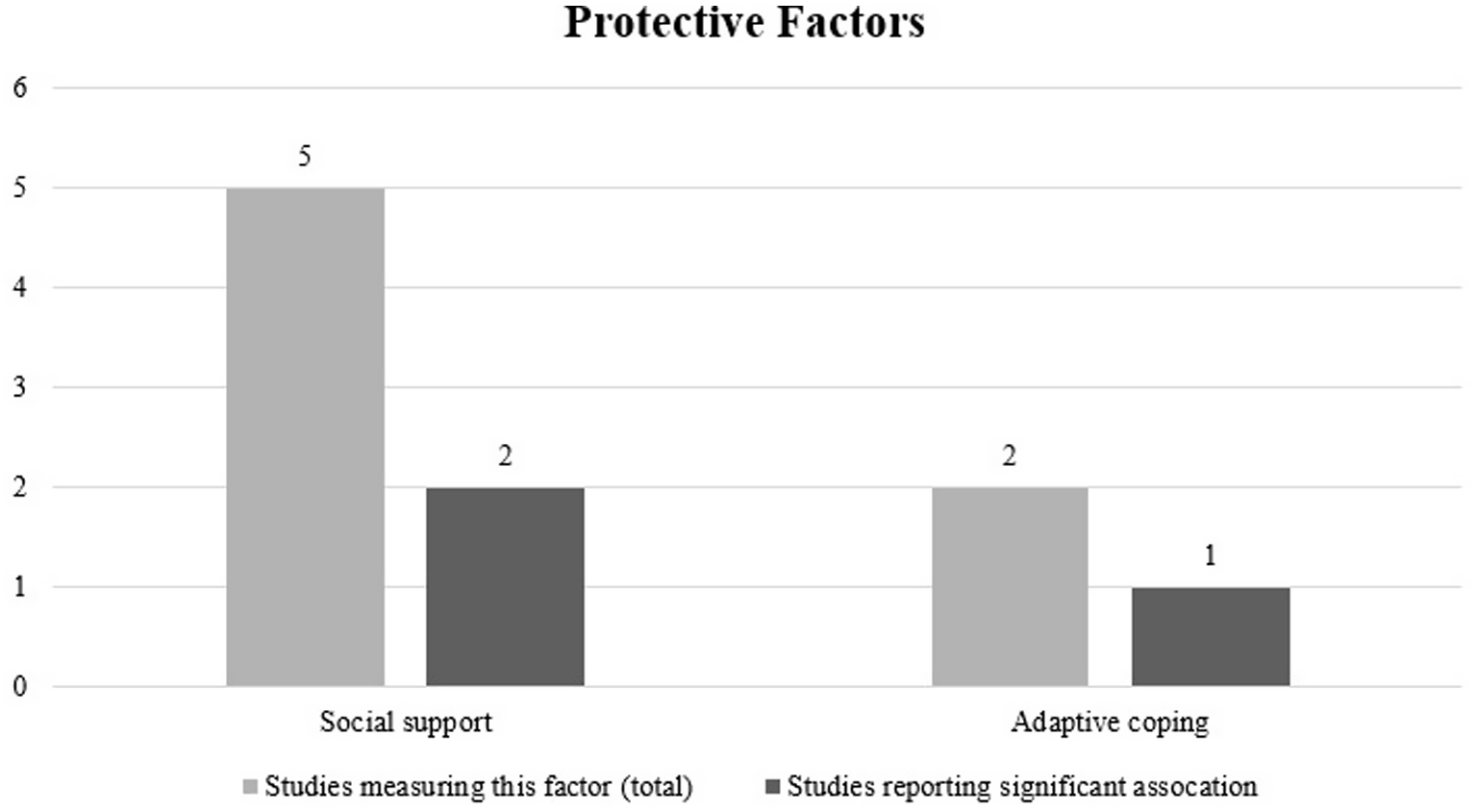
Protective factors for interpersonal revictimization (*N* = 16).

The most commonly studied protective factor against interpersonal revictimization was social support, with two out of five studies indicating that social support was a significant protective factor against interpersonal revictimization (Scoglio et al., 2022; Ullman and Najdowski, 2011) Notably, in the study by Scoglio et al. (2022), social support during military deployment was a significant protective factor, while social support after military deployment was not significantly associated with interpersonal revictimization. One out of two studies demonstrated that adaptive coping was a significant protective factor against revictimization (Iverson et al., 2013). See Figure 4 for an overview of the investigated protective factors.

### 3.5 Risk of bias

**Figure 5:**
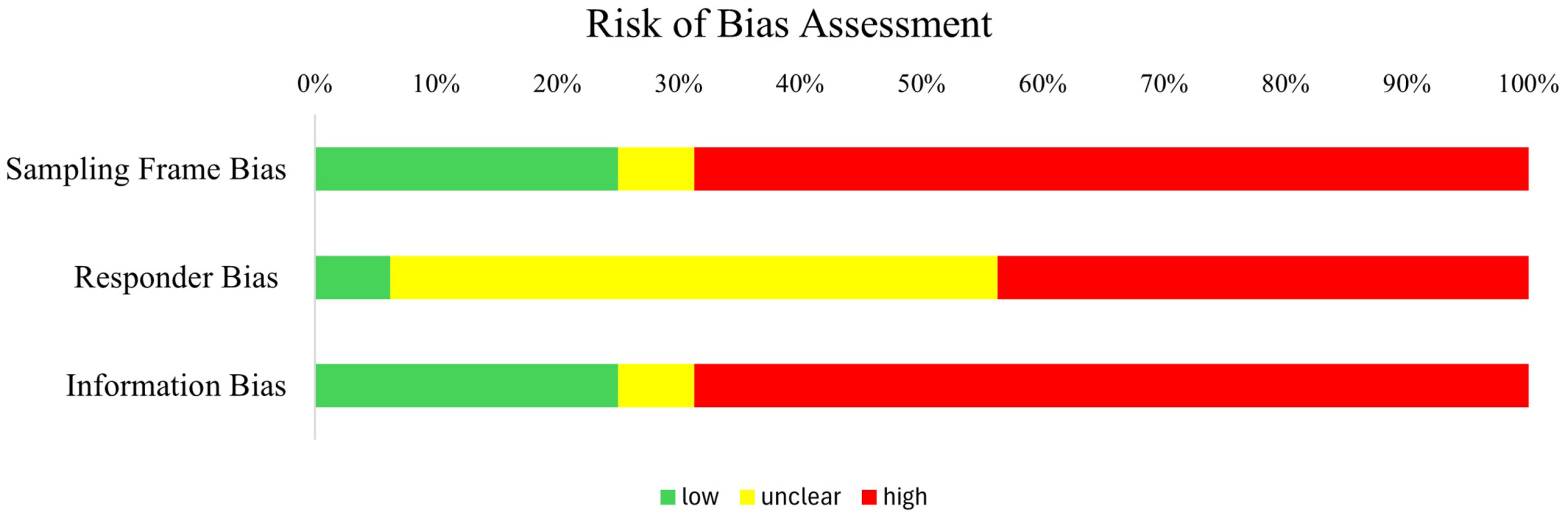
Results of the risk of bias assessment with the RoB-PrevMH tool.

Figure 5 shows that the majority of the included studies score high on both sampling frame bias and information bias. This indicates that in the majority of studies, the study population was not representative of the target population and that the instruments used for the assessment of revictimization were not validated. Moreover, for half of all included studies there was not enough information to judge the risk of responder bias (i.e., what percentage of all people approached consented to participate).

## 4 Discussion

With this systematic review, we aimed to provide an overview of (a) which facets of PTSD (overall severity/symptom clusters) are risk factors for interpersonal revictimization and (b) which non-PTSD factors are related to interpersonal revictimization in the context of PTSD symptoms. To this end, we included 16 longitudinal studies. Our results indicated that the overall severity of PTSD symptoms was a robust risk factor, while the evidence remained mixed for hyperarousal and negative changes in mood and cognition. In this case, robust means that the majority of studies found evidence for the relationship and mixed evidence indicates that some studies found an effect and others did not but there was no majority either way. Interestingly, we found no evidence that intrusion, avoidance, or dissociation were risk factors for interpersonal revictimization. The most robust non-PTSD risk factors were the severity of prior victimization and drug use. The results remained mixed for problematic alcohol use, childhood maltreatment, depression and maladaptive coping. Also for the protective factors, social support and adaptive coping, the results remained mixed. Our results are largely in line with other systematic reviews that examined revictimization after childhood maltreatment/sexual abuse (Fereidooni et al., 2023b; Scoglio et al., 2019; Walker et al., 2019; Walker and Wamser-Nanney, 2023). Given the variability in the types of samples and interpersonal violence under investigation in the current study, we cannot draw definitive conclusions on the effect of these factors on the risk of interpersonal revictimization.

### 4.1 PTSD risk factors

Our finding that the severity of PTSD symptoms is a risk factor for revictimization provides some support for the post-traumatic stress symptom model and is in line with the evidence-based model of revictimization (Walker and Wamser-Nanney, 2023; Fereidooni et al., 2023a). According to the post-traumatic stress symptom model, multiple pathways may lead from PTSD symptoms to revictimization. Among these pathways are difficulties in emotion regulation, anger, substance use, dissociation, and risky sexual behavior. In the current manuscript, we were only able to examine some of these pathways. We therefore cannot draw firm conclusions about the potential working mechanisms that link the severity of PTSD symptoms to revictimization. Moreover, upon inspection of the studies that did not indicate a relationship between PTSD severity and revictimization, we could not identify any common factors that may explain why these studies did not find an association between PTSD severity and revictimization. Namely, the studies focused on different types of revictimization, used different samples (i.e., population sample versus survivors of IPV), and assessed PTSD symptoms with different clinical interviews. Our mixed results regarding the relations of hyperarousal and negative changes in mood and cognition with revictimization are not in line with the evidence-based model for revictimization, which proposes that these factors are associated with revictimization. Because the number of studies that examined the relationship between these symptoms and revictimization was very small, more studies are needed. Similarly, our results that the symptoms of intrusion, avoidance, and dissociation were not related to revictimization also contrast the evidence-based model for revictimization that has proposed that PTSD symptoms are an important risk factor for revictimization (Fereidooni et al., 2023a). Zooming in on symptoms of dissociation, both the evidence-based model for revictimization and the post-traumatic stress symptom model postulate that dissociation is a risk factor for revictimization. Specifically, the evidence-based model for revictimization proposes that peri-traumatic dissociation, i.e., dissociation during the traumatic event, is related to revictimization. However, the studies in this systematic review examined current dissociative symptoms that were assessed after exposure to interpersonal victimization. Therefore, the specific type of dissociative symptoms under investigation may influence whether dissociative symptoms constitute a risk factor for revictimization. Moreover, the authors of the post-traumatic stress symptom model indicated that there was a lack of longitudinal studies that examine the relationship between dissociation and sexual/IPV revictimization (Walker and Wamser-Nanney, 2023). Precisely these types of revictimization incidents are overrepresented in the current manuscript. Therefore, our findings may fill a knowledge gap in the post-traumatic stress symptom model and may be considered as an extension thereof.

### 4.2 Non-PTSD factors

Our findings that the severity of prior victimization and childhood maltreatment were risk factors for revictimization are in line with BTT, the post-traumatic stress symptom model, and the evidence-based model for revictimization (Freyd, 1994; Fereidooni et al., 2023a; Walker and Wamser-Nanney, 2023). Our finding that drug use was a risk factor for revictimization is in line with the post-traumatic stress symptom model and the evidence-based model for revictimization (Fereidooni et al., 2023a; Walker and Wamser-Nanney, 2023). At the same time, the mixed evidence for problematic alcohol use, maladaptive coping, and depression as risk factors for revictimization is not in line with these models (Walker et al., 2019; Fereidooni et al., 2023a). However, in the context of PTSD symptoms, only two studies examined depression and maladaptive coping as risk factors for revictimization. Therefore, we cannot draw firm conclusions about these relationships and more longitudinal studies that examine depression and maladaptive coping as risk factors for revictimization in the context of PTSD symptoms are needed. Lastly, our result that problematic alcohol use was a risk factor for revictimization in one out of four studies contradicts the post-traumatic stress symptom model; however, it corresponds with previous systematic reviews (Walker and Wamser-Nanney, 2023; Scoglio et al., 2019; Fereidooni et al., 2023b). In contrast to our expectations, we found very limited evidence for the protective effects of social support and adaptive coping. A previous systematic review also found little evidence for the protective effects of social support (Fereidooni et al., 2023b). This finding is relevant in the context of the evidence-based model of revictimization that has identified loneliness as an important mediator in the relationship between childhood maltreatment and revictimization (Fereidooni et al., 2023a). More research is needed on the differential effects of loneliness versus (lack of) social support on revictimization to gain a more nuanced understanding of their interrelationships. Regarding the role of adaptive coping, heterogeneity in the definitions of adaptive coping in the included studies may have led to the inconclusive result.

### 4.3 Limitations

The majority of studies included in this review focused on female samples, with only three studies including mixed samples, limiting the generalizability of our findings. The overrepresentation of females may also have influenced the types of interpersonal violence that were reported, as women are more likely than men to be the victim of sexual violence/IPV (Planty et al., 2013; Borumandnia et al., 2020; Bellot et al., 2024). Moreover, most of the studies used either convenience samples, women staying at battered women shelters, or veteran samples. Our findings therefore mainly apply to these specific populations and may have limited applicability for the general or clinical populations. Additionally, the inclusion of samples consisting solely of women staying in shelters may have skewed the results towards more severe and/or frequent revictimization. Most studies focused on IPV as a specific type of interpersonal violence. Given the nature of the intimate relationship between victim and perpetrator, our findings may be mostly applicable to interpersonal violence in the context of an intimate relationship. High variability in the measurement and operationalization of PTSD (diagnosis/overall severity/symptom clusters/based on different diagnostic systems) may have influenced our results and diminished some of our ability to detect the relationship between PTSD symptoms and interpersonal revictimization. However, this relationship still emerged robustly in the current review, underlining the possible strength of the association between PTSD symptoms and interpersonal revictimization. Within the current study, we were not able to examine potential links between revictimization and complex PTSD: a classification in the international classification of disorders—11th edition (ICD-11), which consists of all the symptoms of PTSD plus difficulties in emotion regulation and interpersonal relationships, and distorted beliefs about the self (Cloitre et al., 2019). However, some of the symptoms of complex PTSD, such as the difficulties in emotion regulation and interpersonal relationships, may put people at higher risk for revictimization (Dietrich, 2007). Additionally, given our focus on longitudinal studies in which the assessment of PTSD (symptoms) preceded assessment of revictimization, we cannot draw any conclusions about revictimization as a potential risk factor for PTSD symptoms. Moreover, the high variability in the measurement and operationalization of revictimization reflects the lack of a clear definition of the construct revictimization. This was amongst others, reflected in the observation that although the most commonly used instrument to assess revictimization was the CTS-2, the instrument was often adapted or studies only used subscales of the instrument. These study-specific adaptations make comparisons across studies very difficult. The heterogeneity in measures and operationalizations used was further underlined by the high risk of information bias, which indicates the use of non-validated questionnaires. We furthermore concluded that there was a high risk of responder bias and sampling frame bias. A detailed analysis of methodological features in studies reporting null findings and/or high risk of bias is beyond the scope of the current manuscript and may constrain interpretation of the reported findings. While one of the strong points of the current systematic review is the exclusive focus on longitudinal studies, this may also have introduced bias due to drop-outs in the included studies that may not have been completely at random. There was considerable variability in whether and how studies explicitly addressed missing data. Therefore, based on the included studies and reported results, we cannot exclude the possibility that participants with, for example, higher PTSD symptoms discontinued their participation in the studies. This may have led to an underestimation of the relationships between exposure to risk factors and revictimization. Moreover, the use of longitudinal studies allows the determination of temporal precedence (exposure to risk/protective factor precedes revictimization incident); however, no causal inferences can be drawn based on the current studies. Causal inferences are challenging in this field since randomization of revictimization is ethically impossible. However, future studies may implement structural equation modelling to examine the complex interrelationships between PTSD symptoms, other risk factors, and revictimization (for an example see Ullman and Vasquez, 2015).

### 4.4 Future directions

Only one other systematic review examined the role of social support/depression in the context of revictimization, underlining the need for future studies that examine the role of these factors for revictimization (Fereidooni et al., 2023b). The severity of previous interpersonal violence emerged as a risk factor for revictimization in slightly more than half of the studies. More research needs to be done to clarify the role of the severity of previous interpersonal violence for revictimization as it may be influenced by the developmental period, perpetrator, or social context in which it occurred. Additionally, future research should focus on protective factors against revictimization since they will be crucial for the development of interventions aimed at preventing revictimization. Specific protective factors that may be researched are perceived and received social support, emotion regulation strategies (adaptive versus maladaptive), receiving psychological care, or setting boundaries. Additionally, definitions of revictimization varied greatly across studies and no validated questionnaire exists that specifically assesses revictimization. Therefore, future research should aim to establish a clear definition of revictimization and develop a method of assessment that can be validated with input from clinicians, researchers, and experts by experience. Moreover, we noticed great variability in the assessment of PTSD symptoms. This variability makes the comparison of estimates of the severity of PTSD symptoms across studies difficult. We therefore advise future research to use the golden-standard for screening and assessment of PTSD symptoms, such as the PTSD Checklist for DSM-5 (PCL-5) or the clinician administered PTSD scale for DSM-5 (CAPS-5) (Blevins et al., 2015; Weathers et al., 2018). The employment of these instruments also facilitates the comparison of estimates in the general population with clinical populations, as the PCL-5 and CAPS-5 are routinely used in clinical settings. Research on revictimization is still largely focused on female samples that consist either largely of college women or battered women currently staying in shelters. These populations are not representative of the general population or of clinical populations, which is why we call for more studies using mixed samples and clinical samples. The use of clinical samples seems adequate when taking into account that the severity of PTSD symptoms emerged as the most robust risk factor for interpersonal revictimization. At the same time, the results for the PTSD symptom clusters still remain mixed which is why future research should continue to explore their unique contributions in multivariate models that include all symptom clusters. Moreover, most studies focused on IPV or sexual violence as revictimization incidents, leading to an overrepresentation of these types of interpersonal violence in the literature. However, it has been established that non-sexual (i.e., physical/emotional) interpersonal violence can lead to comparable negative consequences as sexual violence, which is why studies should broaden their focus on more types of interpersonal violence (Schnittker, 2022). Given that sexual violence disproportionally affects women, expanding the focus on more types of interpersonal violence may also make recruitment of mixed samples more feasible (Planty et al., 2013; Borumandnia et al., 2020).

### 4.5 Implications for practice and research

The current study is the first systematic review on interpersonal revictimization with both (a) a focus on PTSD symptoms, and (b) an exclusive focus on longitudinal studies. While we recognize the importance of also providing an overview of cross-sectional evidence in the literature, our exclusive focus on longitudinal studies lends itself best to draw conclusions about risk and protective factors since these terms imply a measure of temporal precedence (i.e., exposure to a risk/protective factor pre-cedes the revictimization). We therefore call for both (a) more longitudinal studies on risk and protective factors for interpersonal revictimization and (b) a clear focus in systematic reviews on longitudinal evidence or the clear differentiation between the cross-sectional and longitudinal evidence in one systematic review (see Fereidooni et al., 2023b for an example of the latter). The current findings are relevant for clinical practice since the severity of PTSD symptoms emerged as the most robust risk factor for interpersonal revictimization. Therefore, screening for and treatment of PTSD symptoms may be a starting point in the prevention of interpersonal revictimization. Besides intervention for PTSD symptoms, it remains important to treat full-blown PTSD to potentially break the cycle of (re-)victimization and PTSD. Additionally, our findings can help increase awareness of the heightened risk of revictimization for people with higher PTSD (symptom) severity among researchers and clinicians. Specifically, clinicians who work with people experiencing (symptoms of) PTSD should pay close attention to the potential for future revictimization during treatment and in relapse-prevention. Researchers should focus on validated and standardized assessment of both PTSD symptoms and revictimization. Lastly, the results of the current study provide an overview of the factors that could be addressed in interventions to prevent interpersonal revictimization (i.e., PTSD symptoms and drug use).

## Data Availability

The original contributions presented in the study are included in the article/Supplementary material, further inquiries can be directed to the corresponding author.
