# Supplementary material for: Risk and protective factors for interpersonal revictimization in people with post-traumatic stress symptoms: a systematic review
Source: Front Psychol. 2025 Dec 10;16:1610030. doi: 10.3389/fpsyg.2025.1610030 (PMC12727439; doi:10.3389/fpsyg.2025.1610030)
Supplement: Supplementary file 2 [file Table_2.docx]

Supplementary 2. Overview of PTSD and Revictimization Assessment and Victimization Status of the Included Samples.

| First Author (Year) | Instrument to assess PTSD (symptoms) | Instrument to assess revictimization | Entire sample victimized at baseline (yes/no) |
| --- | --- | --- | --- |
| Aakvaag et al. (2019) | PTSD Checklist for DSM-IV (PCL-IV; Bliese et al., 2008) | Sexual assault was assessed with four separate questions “Has anyone ever forced you into a) intercourse, b) oral sex, c) anal sex, or d) put fingers or objects into your vagina or anus by use of physical force or by threatening to hurt you or someone close to you?”. Physical assault was assessed with questions from the NSES-W (Kilpatrick et al., 2011), and controlling behavior was assessed with questions adapted from Tolman, 1999. | Yes, all participants experienced some form of childhood violence as reported at baseline. |
| Amstadter et al. (2011) | Structured clinical interview that assesses DSM-IV criteria (Kilpatrick et al., 2000) | Behaviorally specific questions that pertained to having been a) attacked or threatened with a gun, knife, or some other weapon b) attacked by another person with perceived intent to kill or seriously injure, or c) beaten or injured by another person. | No, within the study a subsample that had experienced previous interpersonal violence was selected. |
| Babcock & DePrince (2013) | Posttraumatic Diagnostic Scale (PDS; Foa et al., 1997) | Conflict Tactics Scale - Revised CTS-R; Straus et al., 1996) | Yes, all participants had experienced intimate partner violence (IPV) at baseline. |
| Cascardi & Avery-Leaf, (2019) | PTSD Symptom Severity Scale (PSSS; Foa et al., 1993) | Conflict in Adolescent Dating Relationships Inventory (CADRI; Wolfe et al., 2001) | No, participants reported on their history of IPV and were grouped accordingly. |
| Cole et al. (2008) | Diagnostic Interview Schedule (DIS; Robins et al., 1981) | Questions adapted from other studies on protective orders (Harrell, 1993), from the Conflict Tactics Scale (CTS (Straus, 1995) and CTS-R (Straus et al., 1996), and Toman’s Psychological Maltreatment of Women Inventory (PMWI; Tolman et al., 1999) | Yes, all participants had experienced IPV at baseline. |
| Dardis et al. (2018) | PTSD Checklist-5 (PCL-5; Weathers et al., 2013) | CTS-R (Straus et al., 1996) | Yes, all participants had experienced IPV at baseline. |
| Iverson et al. (2013) | The Posttraumatic Diagnostic Scale (PDS; Foa et al., 1997) | Physical assault subscale of the CTS-R (Straus et al., 1996) | Yes, all participants had experienced IPV at baseline. |
| Iverson et al. (2022) | PTSD Checklist-5 (PCL-5; Bovin et al., 2016; Weathers et al., 2022) | Adapted questions about sexual violence, physical violence, and psychological violence that were adapted from the Lifetime Trauma Interview for Intimate Partner Violence Survivors (Galovski et al., 2019) | No, participants reported on their history of IPV at baseline. |
| Jaffe et al. (2019) | PTSD Checklist-Civilian Version (PCL5-C; Weathers et al., 1993) | Life Events Checklist (LEC; Gray et al., 2004) | No, participants reported on their history of interpersonal trauma at baseline. |
| Krause et al. (2006) | PTSD Checklist-Civilian Version (PCL-IV; Weathers et al., 1993) | Physical and sexual assault subscales of the CTS-R (Straus et al., 1996) | Yes, all participants had experienced IPV at baseline. |
| Kuijpers et al. (2012) | PTSD Symptom Scale-Self Report (PSS-SR; Foa et al., 1993) | Dichotomous version of the psychological and physical assault subscales of the CTS-R (Straus et al., 1996) | Yes, all participants had experienced IPV at baseline. |
| Perez et al. (2012) | The Clinician Administered PTSD scale (CAPS-IV; Blake et al., 1995) | CTS-R (Straus et al., 1996) | Yes, all participant had experienced IPV at baseline. |
| Perez & Johnson (2008) | PTSD Symptom Scale Interview (PSS-I; Foa et al., 1993) | Participants were provided with a blank calendar, representing the past year. They filled in important events, such as a new job, etc, together with a researcher. Subsequently they added violent incidents, if they experienced any. | Yes, all participants had experienced IPV at baseline. |
| Scoglio et al. (2022) | PTSD Checklist-Civilian Version (PCL-C; Weathers et al., 1993) | Two items from the Deployment Risk and Resilience Inventory (DRRI; King et al., 2006) | No, participants reported on their history of childhood abuse, military sexual assault (adulthood), and physical assault (adulthood) at baseline. |
| Stockdale et al. (2014) | Diagnostic Interview Schedule (DIS; Robins et al., 1981) | An adapted version of the Sexual Experiences Questionnaire (SEQ-DoD; Fitzgerald et al., 1999) | Yes, all participants had experienced IPV at baseline. |
| Ullman & Najdowski (2011) | Posttraumatic Stress Diagnostic Scale (PDS; Foa, 1995) | Sexual Experiences Survey (SES; Koss et al., 1987) | Yes, all participants had experienced sexual assault at baseline. |

Note.

**References**

Blake, D. D., Weathers, F. W., Nagy, L. M., Kaloupek, D. G., Gusman, F. D., Charney, D. S., & Keane, T. M. (1995). The development of a clinician-administered PTSD scale*. Journal of Traumatic Stress, 8(1),* 75–90. https://doi.org/10.1007/BF02105408

Bliese, P. D., Wright, K. M., Adler, A. B., Cabrera, O. A., Castro, C. A., & Hoge, C. W. (2008). Validating the primary care posttraumatic stress disorder screen and the posttraumatic stress disorder checklist with soldiers returning from combat*. Journal of Consulting and Clinical Psychology, 76(2),* 272–281. https://doi.org/10.1037/0022-006X.76.2.272

Bovin, M. J., Marx, B. P., Weathers, F. W., Gallagher, M. W., Rodriguez, P., Schnurr, P. P., & Keane, T. M. (2016). Psychometric properties of the PTSD Checklist for Diagnostic and Statistical Manual of Mental Disorders–Fifth Edition (PCL-5) in veterans. *Psychological Assessment, 28(11),* 1379–1391. https://doi.org/10.1037/pas0000254

Fitzgerald, L. F., Magley, V. J., Drasgow, F., & Waldo, C. R. (1999). Measuring Sexual Harassment in the Military: The Sexual Experiences Questionnaire (SEQ—DoD). *Military Psychology*, *11*(3), 243-263. <https://doi.org/10.1207/s15327876mp1103_3>

Foa, E. B. (1995). *Posttraumatic Stress Diagnostic Scale manual*. National Computer Systems.

Foa, E. B., Cashman, L., Jaycox, L., & Perry, K. (1997). The validation of a self-report measure of posttraumatic stress disorder: The Posttraumatic Diagnostic Scale. *Psychological Assessment, 9(4)*, 445–451. https://doi.org/10.1037/1040-3590.9.4.445

Foa, E. B., Riggs, D. S., Dancu, C. V., & Rothbaum, B. O. (1993). Reliability and validity of a brief instrument for assessing post-traumatic stress disorder. *Journal of Traumatic Stress, 6(4),* 459–473. <https://doi.org/10.1002/jts.2490060405>

Galovski, T.; Iverson, K. Lifetime Trauma Interview for Intimate Partner Violence Survivors. 2019, unpublished.

Gray, M. J., Litz, B. T., Hsu, J. L., & Lombardo, T. W. (2004). Psychometric properties of the Life Events Checklist. *Assessment*, *11*, 330–341. https://doi.org/10.1177/1073191104269954

Harrell, A., Smith, B., Newmark, L. (1993). Court processing and the effects of restraining orders for domestic violence victims. *Bureau of Justice Analysis: Institute for Social Analysis; Washington DC*.

King, L. A., King, D. W., Vogt, D. S., Knight, J., & Samper, R. E. (2006). Deployment Risk and Resilience Inventory: A Collection of Measures for Studying Deployment-Related Experiences of Military Personnel and Veterans. *Military Psychology*, *18*(2), 89-120. <https://doi.org/10.1207/s15327876mp1802_1>

Kilpatrick, D. G., Resnick, H. S., Baber, B., Guille, C., & Gros, K. (2011). *The national stressful events web survey (NSES-W)*. Charleston, SC:Medical University of SouthCarolina

Koss, M. P., Gidycz, C. A., & Wisniewski, N. (1987). The scope of rape: Incidence and prevalence of sexual aggression and victimization in a national sample of students in higher education. *Journal of Consulting and Clinical Psychology, 55*, 162-170.

Robins, L. N., Helzer, J. E., Croughan, J. L., & Ratcliff, K. S. (1981). National Institute of Mental Health Diagnostic Interview Schedule: Its history, characteristics, and validity. *Archives of General Psychiatry, 38(4),* 381–389. https://doi.org/10.1001/archpsyc.1981.01780290015001

Straus, M. (1995). *Manual for the Conflict Tactics Scale*. Family Research Laboratory, University of New Hampshire.

Straus, M. A., Hamby, S. L., Boney-McCoy, S., & Sugarman, D. B. (1996). The revised Conflict Tactics Scales (CTS2): Development and preliminary psychometric data. *Journal of Family Issues*, *17*(3), 283-316. <https://doi.org/10.1177/019251396017003001>

Tolman, R. M. (1999). The validation of the Psychological Maltreatment of Women Inventory. *Violence Vict*, *14*(1), 25-37.

Weathers, F. W., Litz, B. T., Herman, D. S., Huska, J. A., & Keane, T. M. (1993, October). *The PTSD Checklist: Reliability, validity, and diagnostic utility*. In *Annual Meeting of the International Society for Traumatic Stress Studies,* San Antonio, TX.

Weathers, F. W., Litz, B. T., Keane, T. M., Palmieri, P. A., Marx, B. P., & Schnurr, P. P. (2013). *The PTSD Checklist for DSM-5 (PCL-5).* National Center for PTSD. <https://www.ptsd.va.gov>

Weathers, F.W.; Litz, B.T.; Keane, T.M.; Palmieri, P.A.; Marx, B.P.; Schnurr, P.P. The PTSD

Checklist for DSM-5 (PCL-5). Available online: http://www.ptsd.va.gov (accessed on 10 April 2022).

Wolfe, D. A., Scott, K., Reitzel-Jaffe, D., Wekerle, C., Grasley, C., & Straatman, A. L. (2001). Development and validation of the conflict in adolescent dating relationships inventory. *Psychological Assessment, 13*(2), 277􂚶293. doi:10.1037/1040 3590.13.2.277
